# A Mixed-Effects Model with Different Strategies for Modeling Volume in *Cunninghamia lanceolata* Plantations

**DOI:** 10.1371/journal.pone.0140095

**Published:** 2015-10-07

**Authors:** Mei Guangyi, Sun Yujun, Xu Hao, Sergio de-Miguel

**Affiliations:** 1 Laboratory for Silviculture and Conservation, Beijing Forestry University, 35 Qinghua East Road, Beijing, China; 2 Faculty of Science and Forestry, University of Eastern Finland, P.O., Joensuu, Finland; 3 Departament de Producció Vegetal i Ciència Forestal, Universitat de Lleida-Agrotecnio Center (UdL-Agrotecnio), Av. Rovira Roure, 191, E–25198, Lleida, Spain; Chinese Academy of Forestry, CHINA

## Abstract

A systematic evaluation of nonlinear mixed-effect taper models for volume prediction was performed. Of 21 taper equations with fewer than 5 parameters each, the best 4-parameter fixed-effect model according to fitting statistics was then modified by comparing its values for the parameters total height (H), diameter at breast height (DBH), and aboveground height (h) to modeling data. Seven alternative prediction strategies were compared using the best new equation in the absence of calibration data, which is often unavailable in forestry practice. The results of this study suggest that because calibration may sometimes be a realistic option, though it is rarely used in practical applications, one of the best strategies for improving the accuracy of volume prediction is the strategy with 7 calculated total heights of 3, 6 and 9 trees in the largest, smallest and medium-size categories, respectively. We cannot use the average trees or dominant trees for calculating the random parameter for further predictions. The method described here will allow the user to make the best choices of taper type and the best random-effect calculated strategy for each practical application and situation at tree level.

## Introduction

The ability to describe the stem form of a forest tree is important for practical and theoretical reasons. Foresters require stem profile models for estimating the volume and the value of the whole stem or a part of it [1], to various utilization limits [2, 3]. Such estimates are essential in forest planning, for example in evaluating the economics of different management regimes [4]. A theoretical aspect of interest is the relationship between stem form, competition and tree age for individual species, which calls for parameter-parsimonious models that can be used to make general statements about the effect of silviculture and site conditions on stem form [5, 6].

Taper models can be classified into simple polynomial, segmented and variable-form models [7]. A comparison of these 3 types of models shows that although the simple polynomial taper models have a notably simple structure and easy convergence, they are not good at accurately describing the stem. The segmented taper models are more complicated and have good accuracy but are also more difficult to calculate. The variable-form taper models have good structure, can accurately predict the stand volume, and are not overly complicated to calculate [8–10]. Therefore, in contrast to the single taper and segmented taper models, the variable-form taper model is widely used.

Because the data for stem taper have hierarchy and repeated measurement [11], many researchers use Nonlinear Mixed-Effects (NLME) models to develop taper models. Compared with the regression method, NLME models consist of fixed- and random-effect parameters and have the advantage of enabling the modeling of the covariance matrix of correlated data. There are 2 responsible variable components in the variance-covariance matrix: the random-effect component and the within-subject component. Both components can be used to model the heteroskedasticity and autocorrelation of a mixed-effect model [12–14].

However, previous studies have mostly considered the fitting of one variable exponent [15]. Many studies [15–21] used the segmented model of Max and Burkhart (1976) [22]. Furthermore, de-Miguel[4] compared the simple polynomial, segmented and variable-form taper model types using both fixed- and random-effect approaches to predict the volume. Finally, Kozak II with 9 parameters [13] was selected as the best taper model. However, the taper model of Kozak II has 9 parameters and a complicated structure. Therefore, there is no systematic comparison of the aforementioned taper models using fixed- and random-effect approaches that has both simple structure and good accuracy.

For the mixed-effect model, the high cost of measuring additional upper-stem diameters makes it difficult to calibrate the tree-specific taper functions in forestry practice. To solve this problem, de-Miguel [23] compared 3 different prediction methods in model evaluation and validation: (1) a fixed-effect model, (2) the fixed part of a mixed-effect model, and (3) Monte Carlo simulation based on a randomized mixed-effect model. Their results suggest that fixed-effect models should be used when the purpose of the model is prediction and calibration data are not available. Crecente-Campogeneralized the NLME height—diameter model for *Eucalyptus globulus* L. in northwestern Spain [24]; random parameters for particular plots were estimated with different tree selections (5 options). Finally, the height—diameter relationships for individual plots were obtained by calibrating the height measurements of the 3 smallest trees in a plot.

First, this study aimed to perform a consistent analysis of the performance of taper models with fewer than 5 parameters and to modify each of them for good accuracy. Using the best model that was found, 7 strategies were compared for volume prediction using a taper model in the absence of additional measurements for tree-specific calibration [25].

## Materials and Methods

### Materials

The study area is in Jiangle state-own forest farm in Fujian province,China. Jiangle state-owned forest farm provide the permission for each location. This forest farm has a compartment as a study area, and these forest lands are all experimental plantation, this is a place for Beijing Forestry University and Jiangle state-owned forest farm for forestry research. Jiangle state-owned forest farm is a place for Chinese fir wood production, the forest area is very big, study the taper is good for the wood trading. And this place has no specific permissions were required for these locations/activities. So we choose this place for case. The main species of the forest farm are Chinese fir, Masson pine, Moso bamboo. Using the data collected from Jiangle state-owned forest farm have published many papers, there has no endangered or protected species. So we confirm that the field studies did not involve endangered or protected species. The region is characterized by ferromagnesian (red) soils and has a mean annual precipitation of approximately 1699 mm, a mean annual frost-free season of 287 days, and a mean annual temperature of 18.7°C [26].

**Fig 1:**
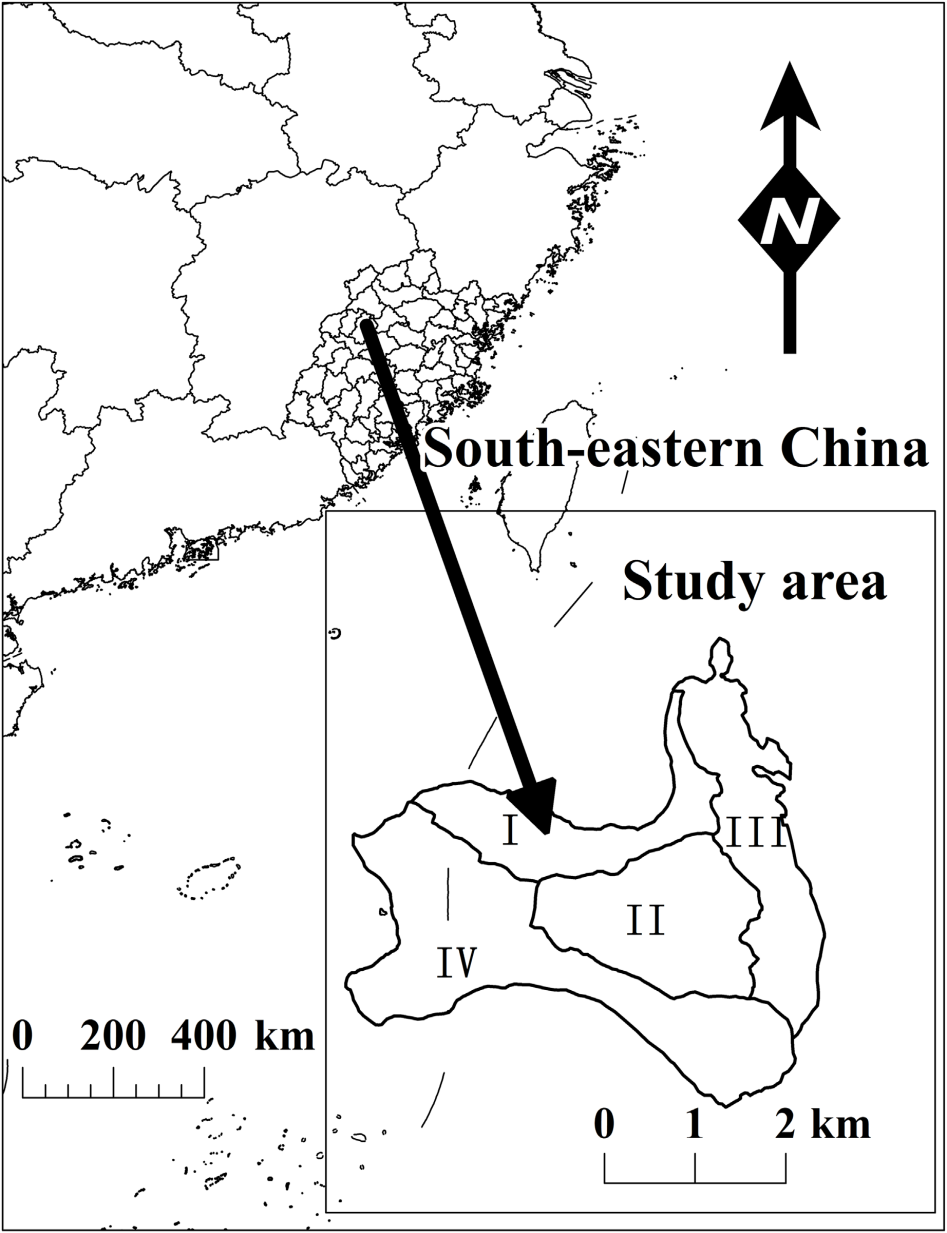
4 sites of Fujian province, Southeast China, where 41 trees were sampled.

**Fig 2:**
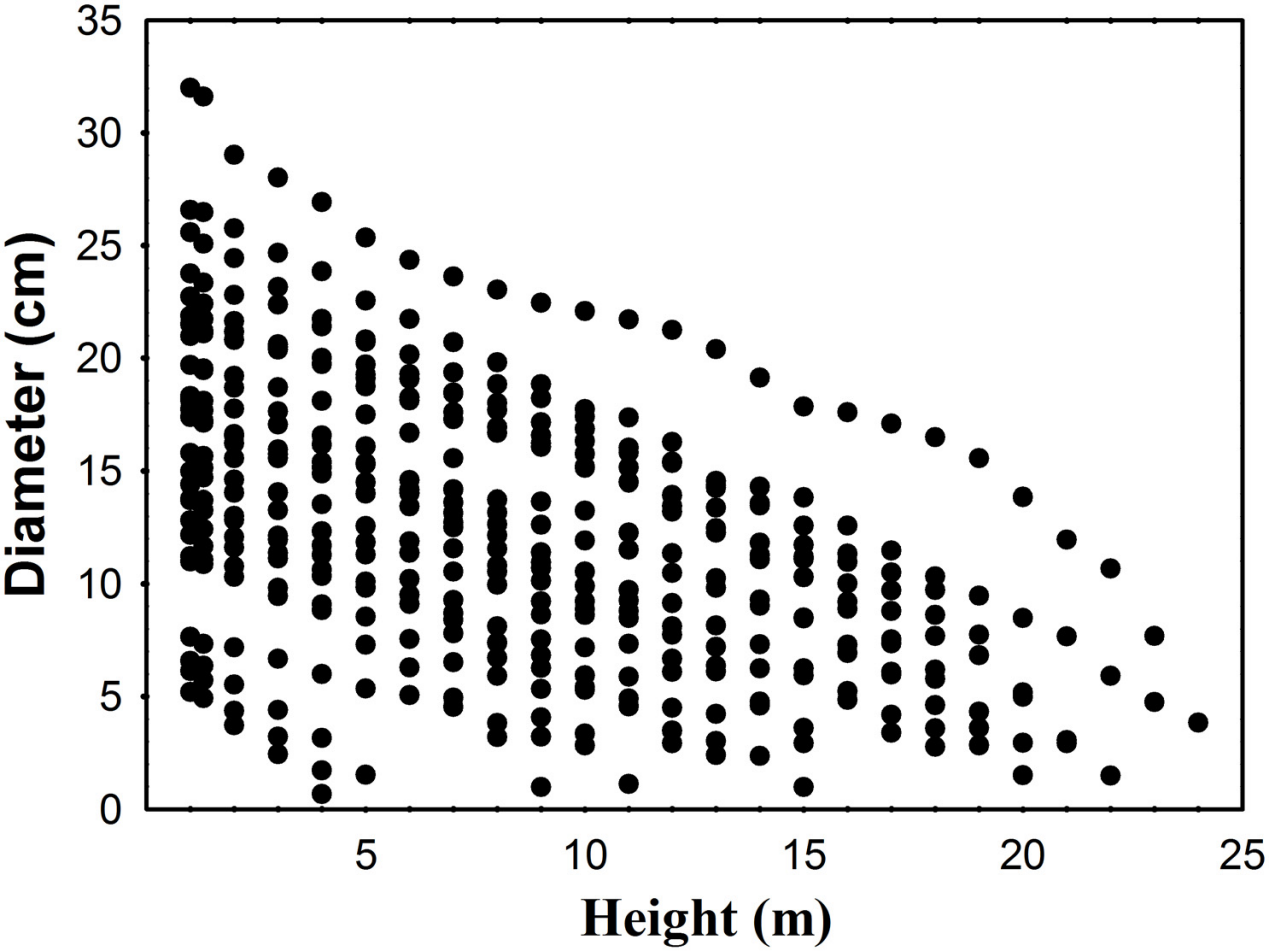
Diameter and total height distribution of the 466 h-d data used from 41 trees to model the taper equations.

We sampled four regions, which were divided equally into 41 plots of *Cunninghamia lanceolata* trees (Qiantan, 15 plots; Shuinan, 8 plots; Yuhua, 11 plots; and Yuandang, 9 plots) and are represented by I, II, III and IV, respectively, in Fig 1. Established between 2010 and 2014, the plots vary in size from 400 to 600 m^2^. In the plots, we measured the diameters at breast height (DBHs) over the bark (at 1.3 m above ground) of fresh trees (height > 1.3 m) and the total tree height of 41 trees that were felled for stem analysis. Before felling each tree, we measured two attributes: diameter at breast height (1.3 m above ground) and total tree height (H). After felling, we measured the diameter at intervals of 1 m and 2 m above the breast height depending on the total tree height. We further performed a laboratory analysis of the outer and inner bark of each disc. These diameters were measured along the largest axis and smallest axis (Table 1 and Fig 2).

**Table 1:** Summary of tree attributes for the *Cunninghamia lanceolate*.

	DBH(cm)	Total height(m)	Disk dob (cm)	Disk height(m)
**Mean**	17.3	17.3	12.0	7.7
**SD**	5.8	5.5	6.0	5.9
**Minimum**	4.9	4.1	0.7	0.0
**Maximum**	28.4	25.5	30.4	25.0

### Selection of candidate equations

The ranking and selection of the taper models were performed in three steps. First, 21 published candidate equations are variable-form taper models with fewer than 5 parameters (Tables 2 and 3) [27]. The best function was selected by applying 5 statistical criteria: Mean Absolute Bias (MAB), root mean square error (RMSE), adjusted coefficient of determination R^2^, Akaike’s information criterion (AIC), and Bayesian information criterion (BIC) [28].

**Table 2:** List of 21 candidate taper models, which were classified according to the number of parameters (NP) developed in this study.

NP	Models evaluated
**1**	Kozak et al. (a) (1969)[31],Ormerod(1973)[32], Demearchalk (a) (1972)[33]
**2**	Kozak et al. (b) (1969)[31],Biging(1984)[34], Newberry and Burkhart (a) (1986)[35], Newberry and Burkhart (b) (1986), Reed and Green[36], Forslund (1990)[37]
**3**	Coffre(1982)[38], Kozak1969(c)[31], Real and Moore (1986)[39], Manuel(2015) [40]
**4**	Bennett and Swindel(1972)[41], Demaerschalk (b) (1972)[33], Demaerschalk (b)(1973)[42],Zeng Weisheng(1997)[43], Sharma(2009)[44], Goulding and Murray (1976)[45]
**5**	Lee et al. (2003)[46], Cervera (1973)[47]

**Table 3:** The 21 candidate equations and their corresponding mathematical expressions.

Eq.	Model	Expression
**1**	Zeng Weisheng (1997)	$d/D=X^{\left(b1+b2z^{0.25}+b3z^{0.5}+b4(D/H)\right)}$
**2**	Sharma (2009)	**$d/D=b1((H-h)/(H-1.37)){\left(H/1.37\right)}^{b2+b3z+b4z^{2}}$**
**3**	Lee (2003)	**$d=b1(D^{b2}){\left(1-z\right)}^{\left(b3z^{2}+b4z+b5\right)}$**
**4**	Ormerod (1973)	*d*/*D* = *X* ^*b*1^
**5**	Demearchalk (a)(1972)	**$d^{2}=(40000/pi)V{\left(H-h\right)}^{(b1-1)b1/H^{b1}}$**
**6**	Cervera (1973)	*d*/*D* = *b*1+*b*2*X*+*b*3*X* ^2^ *+b*4*X* ^3^ *+b*5*X* ^4^
**7**	Goulding and Murray (1976)	*d* ^2^ *KH*/*V*−2*T* = (*b*1(3*T* ^2^−2*T*)+*b*2(4*T* ^3^−2*T*)+*b*3(5*T* ^4^−2*T*)+*b*4(6*T* ^5^−2*T*)
**8**	Real and Moore (1986)	*d* ^2^/*D* ^2^ = *X* ^2^+*b*1(*X* ^3^−*X* ^2^)+*b*2(*X* ^8^−*X* ^2^)+*b*3(*X* ^40^−*X* ^2^)
**9**	Biging (1984)	*d* = *D*(*b*1+*b*2log(1−*z* ^1/3^))(1−exp(−*b*1/*b*2))
**10**	Kozak(a) (1969)	*d* ^2^/*D* ^2^ = *b*1(1−2*z*+*z* ^2^)
**11**	Kozak(b) (1969)	*d* ^2^/*D* ^2^ = *b*1(*z*–1)+*b*2(*z* ^2^−1)
**12**	Kozak(c) (1969)	*d* ^2^/*D* ^2^ = *b*1+*b*2*z*+*b*3*z* ^2^
**13**	Newberry and Burkhart (a)(1986)	*d* = *b*1*D*(*H*−*h*)^*b*2^
**14**	Newberry and Burkhart(b)(1986)	*d* = *b*1*DX* ^*b*2^
**15**	Reed and Green(1984)	*d* ^2^/*D* ^2^ = *b*1*D*(1−*z*)^*b*2^
**16**	Forslund(1990)	*d*/*D* = (1−*z* ^*b*1^)^1/*b*2^
**17**	Demaerschalk (b) (1972)	*d* = *b*1*D* ^*b*2^(*H*−*h*)^*b*3^ *H* ^*b*4^
**18**	Demaerschalk (1973)	*d* ^2^/*D* ^2^ = *b*1(1/(*D* ^2^ *H*))((*H*−*h*)^*b*2^/*H*)+*b*3((*H*−*h*)/*H*)^*b*4^
**19**	Bennett and Swindel (1972)	*d*/*D = b*1*X*+*b*2*W/D+b*3*WH/D+b*4*W*(*H*+*h*+1.3)/*D*
**20**	Coffre(1982)	*d* ^2^/*D* ^2^ = *b*1*X+b*2*X* ^2^ *+b*3*X* ^3^
**21**	Manuel(2015)[40]	$d=2\left(\begin{array}{l} \left(b1D\right)/\left(1-\text{exp}\left(b3S\right)\right)+\left(D/2-b1D\right)\\ \left(1-\left(1/\left(1-\text{exp}\left(b2S\right)\right)\right)\right)+\left(\text{exp}\left(-b2h\right)\right)\\ \left(\left(\left(D/2-b1D\right)\text{exp}\left(1.3b2\right)\right)/\left(1-\text{exp}\left(b2S\right)\right)\right)\\ -\text{exp}\left(b3h\right)\left(\left(b1D\text{exp}\left(-b3H\right)\right)/\left(1-\text{exp}\left(b3S\right)\right)\right) \end{array}\right)$

Note: V = 0.00005806*(D^1.955335^)*(H^0.894033^); W = (H-h)*(h–1.3); T = (H-h)/(H); z = h/H; K = π/40000;S = 1.3-H; X = ((H-h)/(H–1.3)); H: total height (m); h: height above ground level (m); D: diameter at breast height outside the bark (cm); d: diameter outside the bark at height h (cm); b1, b2, b3, b4, and b5 are parameters.

Second, we modified the best model because building an equation with fewer parameters while maintaining good accuracy in volume prediction was the main goal of this study. The model was modified by comparing the relationships among the total height (H), diameter at breast height (DBH), and height above ground level (h) against the modeling data. The best taper model for d^2^ provides unbiased predictions for the cross-sectional area and volume [29–30]. Therefore, all fitted candidate models used d_ki_ as the ith diameter measurement [4]:

$$d_{ki}^{2}=f(h_{ki};D_{k};H_{k};q)+\varepsilon_{ki}$$ (1)

where d_ki_ is the ith diameter measurement of tree k, which is measured at height h_ki_, D_k_ and H_k_ are the DBH and total height of tree k, respectively, q is the vector of 1–5 parameters, and *ε*_*ki*_ is the residual.

### Volume calculation based on taper model

Based on taper model selected above, Formula (2) was used calculate the volume of trees.

$$\frac{\pi }{40000}\int_{0}^{\text{H}}{\text{d}}^{2}*\text{dh}$$ (2)

where H is the total height, d is the diameter outside the bark at height h (cm), h is the height above ground level.

### Testing different prediction strategies using different random parameters

For the best taper model, the calibrated response was evaluated for different height sampling designs and sampling sizes within all data to calculate the tree random parameter for different heights diameter estimation. Randomly calculate the total height of different number trees from 1 to 10 for random parameter calculation in model calibration and using the remaining trees for validation in 7 strategies. The 7 selected alternatives are as follows:

1. calculating a fixed-parameter model (with no random-effect parameter).
2. calculating the fixed part of a mixed-effect model (random-effect parameter is 0).
3. calculating the heights of the randomly selected trees (total heights of 1–10 randomly selected trees to calculate the parameters).
4. calculating the heights of the largest selected trees (calculating the total heights of 1–10 largest trees to calculate the parameter).
5. calculating the heights of the smallest selected trees (total heights of 1–10 smallest trees to calculate the parameters).
6. calculating the heights of the medium-size selected trees (total heights of 1–10 medium-size trees to calculate the parameters).
7. calculating the heights of a mix of selected trees (calculating the total heights of 3, 6 and 9 trees in the largest, smallest and medium-size categories) [4, 22,24].

## Results

### Analyzing the candidate taper model to select the best model

The performance of 5 stem taper functions shows that the function has the form of ((*H*-*h*)/(*H*–1.3))^*b*0^, except for the model of (16) and (21), which does not have that structure for *Cunninghamia lanceolata*. The Model (1) has the best accuracy (Table 4) and is therefore the best candidate model.

**Table 4:** Fitting statistics for 5 selected models for detailed analyses.

Model	NP	R^2^	RMSE	MAB	ΔAIC	ΔBIC
**Ormerod(1973)**[29]	1	0.965	1.142	0.734	107	94
**Forslund(1990)**[33]	2	0.954	1.315	0.862	341	333
**Manuel(2015)** [48]	3	0.963	1.176	0.809	2805	2800
**Zeng Weisheng(1997)**[38]	4	0.970	1.061	0.677	0	0
**Cervera(1973)**[42]	5	0.969	1.077	0.754	20	24

Note: ΔAIC and ΔBIC represent the difference in AIC or BIC as compared with the best equation. The best model is the one presenting the lowest ΔAIC or ΔBIC.

The comparison of parameter b0 with H, D and h shows that b0 significantly correlates with h, correlates with D and exhibits only a normal relationship with H. The above b0 equation can be rewritten as follows Eq (3):

b0=f(h)=b1+b2(h) (3)

where b0, b1, b2 is the parameter, h is the height above ground level.

**Fig 3:**
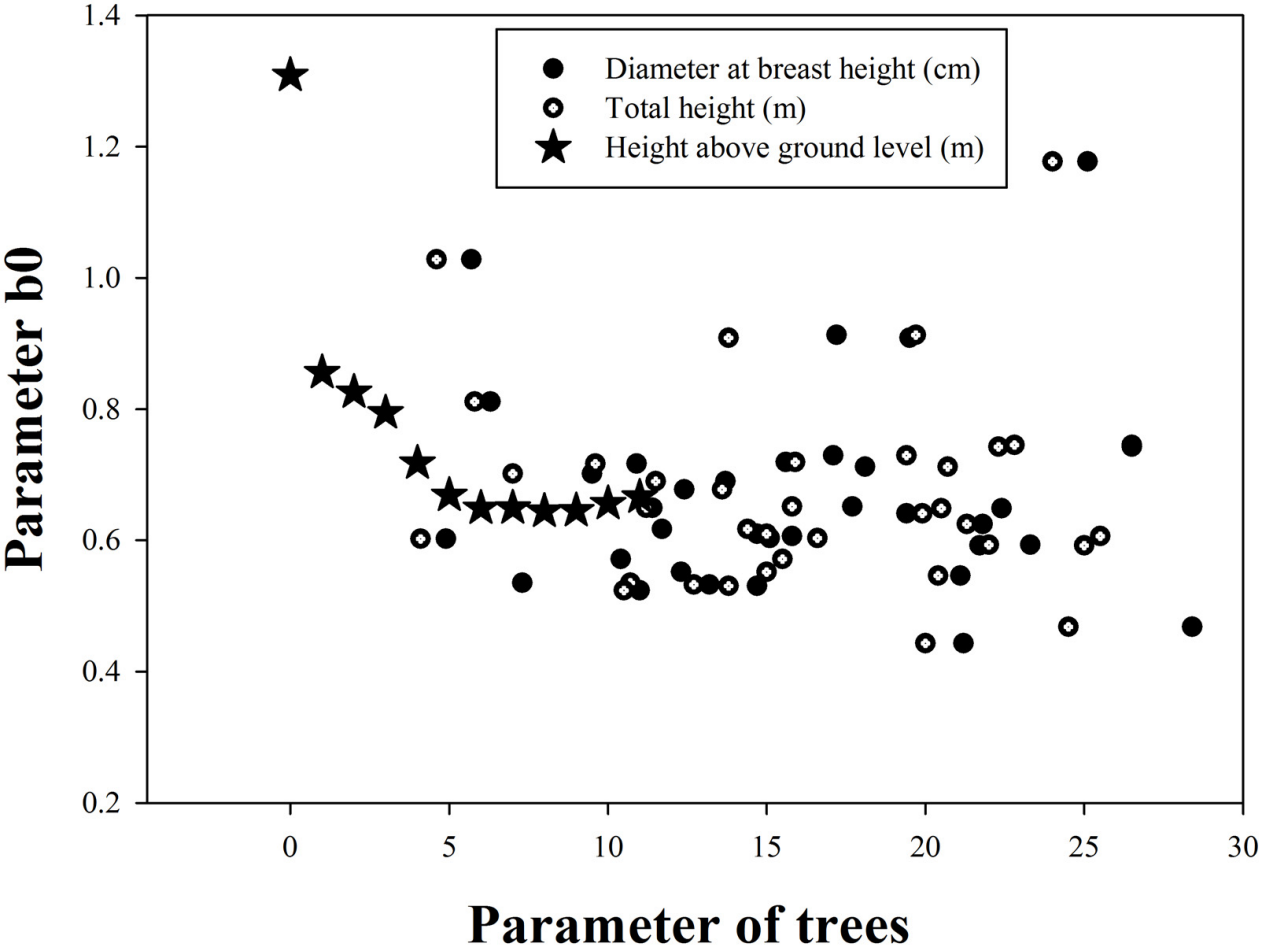
Relationship between the aboveground height, total height, diameter at breast height and the parameter b0.

From the results in Fig 3, we deduce that parameters H, D and b0 are not suitable for increasing the accuracy of the model.

We found that the new taper model with two parameters has a smaller residual than Eq (1) with 4 parameters (Table 5). Because including too many parameters is unsuitable for the model’s convergence, the new taper model is the simplest model that is suitable for volume prediction.

**Table 5:** Comparison between the new taper model and the best taper model.

Models	R^2^	RMSE	MAB	NP
**(1)**	0.970	1.065	0.677	4
**$d^{2}=D^{2}{\left(\frac{\left(H-h\right)}{\left(H-1.3\right)}\right)}^{\left(b1+b2h^{0.007}\right)}$**	0.971	1.048	0.675	2

### A mixed-effect taper model based on the new taper model

Fitting with the NLME function [48] of R using ML was successful with one to three tree-specific random parameters and with a higher number of parameters in some cases. However, we restricted the analysis to the models with one or two random parameters. The model did not converge for parameter (b1, b2) or (b1) with the random parameter β, and the model only converged for (b1) with the random parameter β. Thus, the best model according to the likelihood ratio tests was Formula (4).

$$d^{2}=D^{2}{\left(\frac{\left(H-h\right)}{\left(H-1.3\right)}\right)}^{\left((b1+\beta )+b2h^{0.007}\right)}+\varepsilon_{ki}$$ (4)

where the fixed parameter is b1[44], β is the random parameter, H is the total height, d is the diameter outside the bark at height h (cm), h is the height above ground level, D is the diameter at breast height.

The residual variance was assumed to follow the following Model (5):

$$d(\varepsilon_{i})=\delta^{2}{\left(\delta_{1}+D_{i}{}^{\delta_{2}}\right)}^{2}$$ (5)

where σ^2^ is within-tree residual variance, D_i_ is the ith tree diameter at breast height,δ_1_ and δ_2_ is variance—covariance parameters for random effects.

### Evaluation of the prediction strategies based on the best model

Based on the random parameters for all developing trees, which were estimated to predict the volume, the MAB was 0.0108, the R^2^ was 0.9981, and the RMSE was 0.0119.

Strategie 7 calculated the total height of 3, 6 and 9 trees in the largest, smallest and medium-size categories and obtained the smallest MAB (0.0119), an adjusted coefficient of determination R^2^ of 0.9900, and the smallest RMSE, of 0.0185. Strategies 1, 2 and 5 produced similar values for MAB, RMSE, and R^2^. Strategie 2 calculated the fixed part of a mixed-effect model better than strategie 1, which calculated a fixed-parameter model with MAB 0.0006 m^3^, R^2^ 0.0003, and RMSE 0.0003 m^3^, and strategie 5, which calculated the total height of the 1–10 smallest trees with MAB 0.0001 m^3^, R^2^ 0.0002, and RMSE 0.0002 m^3^. Strategie 3, which calculated the total height of 1–10 randomly selected trees, and strategie 4, which calculated the total height of the 1–10 largest trees, had low accuracy. The worst strategie is strategie 6, which calculated the total height of 1–10 medium-size trees; its R^2^ is only 0.9480, which is substantially poorer than the best R^2^ of 0.0420.

## Discussion

This paper provides a thorough generalization of many published taper equations with fewer than 5 parameters. The accuracy of the models in an independent data set shows that the sample size and design were sufficient [50–51]. In general, taper models with more parameters produce better fit than those with fewer parameters. However, we found that the models with fewer parameters performed better than certain models with more parameters; for example, the equation $d^{2}=D^{2}{\left(\frac{\left(H-h\right)}{\left(H-1.3\right)}\right)}^{\left((b1+\beta )+b2h^{0.007}\right)}$ with two parameters performs better than Eq 1 with four parameters or the equations with five parameters (Table 6). We also know that including too many parameters in nonlinear mixed-effect models is not good for convergence. Thus, under some conditions, the modified model in this paper may be better than other models with more parameters for *Cunninghamia lanceolata* in Fujian Province, China, or for other trees worldwide.

**Table 6:** Estimates of two fixed regression coefficients and the random parameter of the mixed-effect model based on the modified taper model.

Parameters	Estimates	Std.Error	p-value	t-value
**b1**	3.4667	0.1038	<0.001	27.9762
**b2**	-2.1537	0.09153	<0.001	-18.1601
**Var(β)**	0.2399			
**σ** ^**2**^	0.1822			
**δ**_**1**_	17.5855			
**δ**_**2**_	0.8472			

In the taper Model (1), in parameter b0, a larger height corresponds to a smaller b0. Thus, we choose parameter h as the only parameter in b0 to modify the taper, and the validation process demonstrates that this method is accurate.

**Fig 4:**
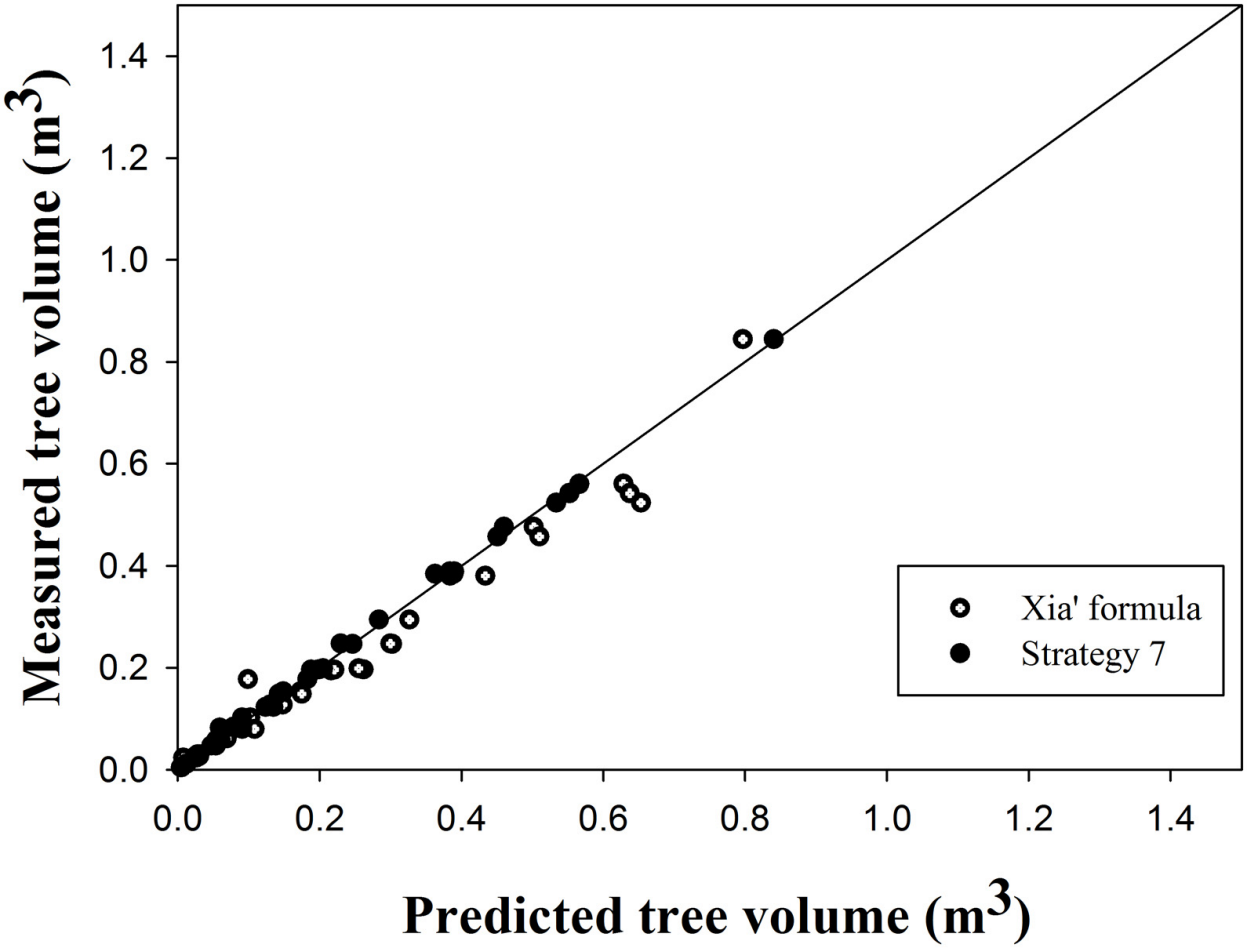
Predictions with the Xia’s volume equations and strategie 7 vs. the measured stem volume [49].

Several strategies were compared in this study using the model that was considered the best based on ease of convergence and a small number of parameters. Seven prediction strategies are readily used in forestry practice. Strategie 7, which calculates the total height of 3, 6 and 9 trees in the largest, smallest and medium-size categories, respectively, has the best accuracy (Fig 4), which suggests that the largest and smallest trees show substantial differences in stem form. The numbers for the 3, 6 and 9 trees large, small and medium-size categories form a nearly normal distribution. Thus, computing the random-effect parameters of the largest, smallest, and medium-size trees clearly improves the predictive accuracy. The calibrated taper model allows the acquisition of accurate results with a notably small sampling effort, which makes this method extremely effective and useful (Table 7).

**Table 7:** Results of model evaluation and validation in volume prediction (m^3^) according to different prediction strategies without calibration.

Data	Strategies	MAB	R^2^	RMSE
**Modeling data**	Strategy 1	0.0121	0.9900	0.0185
	Strategy 2	0.0127	0.9897	0.0188
	Strategy 3	0.0131	0.9881	0.0202
	Strategy 4	0.0138	0.9866	0.0215
	Strategy 5	0.0122	0.9898	0.0187
	Strategy 6	0.0283	0.9480	0.0428
	Strategy 7	0.0119	0.9900	0.0185
**Validation data**	Strategy 1	0.0121	0.9902	0.0171
	Strategy 2	0.0116	0.9915	0.0168
	Strategy 3	0.0133	0.9899	0.0202
	Strategy 4	0.0147	0.9846	0.0222
	Strategy 5	0.0135	0.9874	0.0198
	Strategy 6	0.0246	0.9680	0.0247
	Strategy 7	0.0114	0.9918	0.0163

Strategie 1, which calculates a fixed-parameter model, and strategie 2, which calculates the fixed part of a mixed-effect model, have good accuracy with nearly random parameters for all developing trees. In other words, when the purpose of the model is prediction and calibration data are not available, strategies 1 and 2 should be used based on the best taper model that was modified in this paper. These results are similar to those of de-Miguel [4, 23].

**Fig 5:**
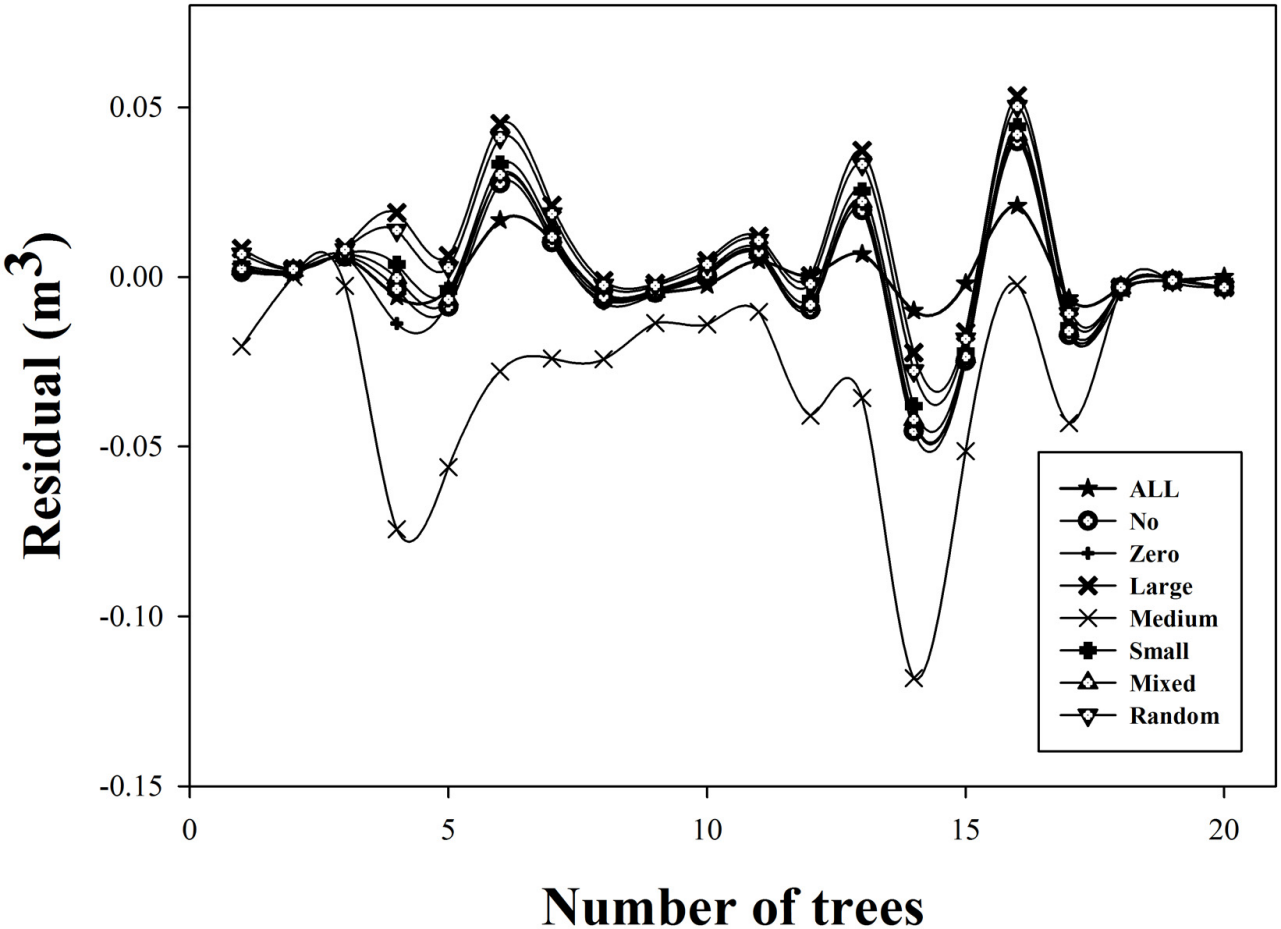
Residuals for the calibrated model with different tree sampling designs and sampling sizes to calculate the random parameters. Note: All: calculate all trees; no: with no random parameter; zero: random parameter is 0; large: largest trees; medium: medium-size trees, small: smallest trees; mixed: a mix of large, medium and small trees; random: randomly selected trees.

Strategies 6 and 4 calculate the heights of medium-size selected trees (total height of 1–10 medium-size trees to calculate the parameters) and the largest selected trees (total height of 1–10 largest trees to calculate the parameters), respectively. Based on the bias results (Fig 5), we find that strategies 6 and 4 were the poorest approaches: strategie 4, using the largest trees, has a larger bias than the other strategies, and strategie 6, using medium-size trees, has a smaller bias than the other strategies. In forest practice, the sample trees are usually average trees, and the medium-size trees are always the average tree in a sample plot (medium-size tree as analytic trees with the average diameter at breast height). Similarly, the largest trees are always the dominant tree in a sample plot, in which they have the largest DBH or total height. Thus, when we use the NLME to predict the volume in a forest stand, we cannot use the average trees or dominant trees to calculate the random parameter to estimate the stand volume; those approaches would produce the lowest accuracy.

## Conclusion

The taper model developed in this paper is the best taper model for describing stands of *Cunninghamia lanceolata*. It has the advantage of easy convergence and simple structure, is a useful tool for predicting the volume of *Cunninghamia lanceolata* and may also be useful for analyzing the taper of other trees worldwide.

In forest practice, when we use the NLME to estimate the stand volume, we cannot use the average trees or dominant trees to calculate the random parameter as the stand random parameter. We should sample some small trees in a mixed approach (strategie 7) to obtain good accuracy.

The results of this study show that when the purpose of the taper model is prediction and calibration data are not available, fixed-effect (with no random parameter) or mixed-effect (random parameter is 0) models should be used. However, because calibration may sometimes be performed for some but not all types of wood, strategie 7 is one of the best strategies to improve the volume prediction accuracy at tree level. This strategie helps the user make the best selection in random-effects calculation for practical applications and scenarios.

## Supporting Information

S1 Text Minimal data set of 35 cut trees. (DOCX)

S2 Text R code for model analysis. (DOCX)
