# Supplementary material for: A Mixed-Effects Model with Different Strategies for Modeling Volume in Cunninghamia lanceolata Plantations
Source: PLoS One. 2015 Oct 7;10(10):e0140095. doi: 10.1371/journal.pone.0140095 (PMC4596836; doi:10.1371/journal.pone.0140095)
Supplement: S1 Text — (DOCX) [file pone.0140095.s001.docx]

Minimal data set of 35 cut trees (total parts of 41)

Note: No is the tree number, h is the height above ground level (m), D is the diameter at breast height outside the bark (cm); d is the diameter outside the bark at height h (cm).

| **No** | **h** | **H** | **D** | **d** |
| --- | --- | --- | --- | --- |
| 1 | 0 | 15.8 | 17.7 | 20.68 |
| 1 | 1 | 15.8 | 17.7 | 18.3 |
| 1 | 1.3 | 15.8 | 17.7 | 17.65 |
| 1 | 2 | 15.8 | 17.7 | 16.53 |
| 1 | 3 | 15.8 | 17.7 | 15.92 |
| 1 | 4 | 15.8 | 17.7 | 15.37 |
| 1 | 5 | 15.8 | 17.7 | 15.33 |
| 1 | 6 | 15.8 | 17.7 | 14.13 |
| 1 | 7 | 15.8 | 17.7 | 13.6 |
| 1 | 8 | 15.8 | 17.7 | 13.15 |
| 1 | 9 | 15.8 | 17.7 | 10.67 |
| 1 | 10 | 15.8 | 17.7 | 8.86 |
| 1 | 11 | 15.8 | 17.7 | 8.46 |
| 1 | 12 | 15.8 | 17.7 | 6.66 |
| 1 | 13 | 15.8 | 17.7 | 6.1 |
| 1 | 14 | 15.8 | 17.7 | 4.58 |
| 1 | 15 | 15.8 | 17.7 | 2.91 |
| 2 | 0 | 10.7 | 7.3 | 9.2 |
| 2 | 1 | 10.7 | 7.3 | 7.63 |
| 2 | 1.3 | 10.7 | 7.3 | 7.31 |
| 2 | 2 | 10.7 | 7.3 | 7.17 |
| 2 | 3 | 10.7 | 7.3 | 6.65 |
| 2 | 4 | 10.7 | 7.3 | 5.97 |
| 2 | 5 | 10.7 | 7.3 | 5.34 |
| 2 | 6 | 10.7 | 7.3 | 5.05 |
| 2 | 7 | 10.7 | 7.3 | 4.52 |
| 2 | 8 | 10.7 | 7.3 | 3.8 |
| 2 | 9 | 10.7 | 7.3 | 3.2 |
| 3 | 0 | 14.4 | 11.7 | 14.98 |
| 3 | 1 | 14.4 | 11.7 | 12.15 |
| 3 | 1.3 | 14.4 | 11.7 | 11.66 |
| 3 | 2 | 14.4 | 11.7 | 11.6 |
| 3 | 3 | 14.4 | 11.7 | 11.1 |
| 3 | 4 | 14.4 | 11.7 | 10.32 |
| 3 | 5 | 14.4 | 11.7 | 9.84 |
| 3 | 6 | 14.4 | 11.7 | 9.5 |
| 3 | 7 | 14.4 | 11.7 | 8.68 |
| 3 | 8 | 14.4 | 11.7 | 7.36 |
| 3 | 9 | 14.4 | 11.7 | 6.82 |
| 3 | 10 | 14.4 | 11.7 | 5.28 |
| 3 | 11 | 14.4 | 11.7 | 4.88 |
| 3 | 12 | 14.4 | 11.7 | 4.48 |
| 3 | 13 | 14.4 | 11.7 | 3 |
| 4 | 0 | 24 | 25.1 | 30.44 |
| 4 | 1 | 24 | 25.1 | 25.56 |
| 4 | 1.3 | 24 | 25.1 | 25.06 |
| 4 | 2 | 24 | 25.1 | 24.42 |
| 4 | 3 | 24 | 25.1 | 23.14 |
| 4 | 4 | 24 | 25.1 | 21.38 |
| 4 | 5 | 24 | 25.1 | 20.8 |
| 4 | 6 | 24 | 25.1 | 19.26 |
| 4 | 7 | 24 | 25.1 | 18.42 |
| 4 | 8 | 24 | 25.1 | 17.68 |
| 4 | 9 | 24 | 25.1 | 16.2 |
| 4 | 10 | 24 | 25.1 | 15.16 |
| 4 | 11 | 24 | 25.1 | 14.46 |
| 4 | 12 | 24 | 25.1 | 13.44 |
| 4 | 13 | 24 | 25.1 | 12.25 |
| 4 | 14 | 24 | 25.1 | 11.27 |
| 4 | 15 | 24 | 25.1 | 10.29 |
| 4 | 16 | 24 | 25.1 | 9.18 |
| 4 | 17 | 24 | 25.1 | 8.78 |
| 4 | 18 | 24 | 25.1 | 7.67 |
| 5 | 0 | 20.5 | 22.4 | 25.26 |
| 5 | 1 | 20.5 | 22.4 | 22.71 |
| 5 | 1.3 | 20.5 | 22.4 | 22.4 |
| 5 | 2 | 20.5 | 22.4 | 21.62 |
| 5 | 3 | 20.5 | 22.4 | 20.35 |
| 5 | 4 | 20.5 | 22.4 | 19.98 |
| 5 | 5 | 20.5 | 22.4 | 19.24 |
| 5 | 6 | 20.5 | 22.4 | 18.11 |
| 5 | 7 | 20.5 | 22.4 | 17.58 |
| 5 | 8 | 20.5 | 22.4 | 16.67 |
| 5 | 9 | 20.5 | 22.4 | 16.05 |
| 5 | 10 | 20.5 | 22.4 | 15.1 |
| 5 | 11 | 20.5 | 22.4 | 14.48 |
| 5 | 12 | 20.5 | 22.4 | 13.18 |
| 5 | 13 | 20.5 | 22.4 | 12.44 |
| 5 | 14 | 20.5 | 22.4 | 11.06 |
| 5 | 15 | 20.5 | 22.4 | 10.28 |
| 5 | 16 | 20.5 | 22.4 | 8.88 |
| 5 | 17 | 20.5 | 22.4 | 7.33 |
| 5 | 18 | 20.5 | 22.4 | 5.76 |
| 5 | 19 | 20.5 | 22.4 | 4.31 |
| 5 | 20 | 20.5 | 22.4 | 2.93 |
| 6 | 0 | 22 | 23.3 | 27.41 |
| 6 | 1 | 22 | 23.3 | 23.74 |
| 6 | 1.3 | 22 | 23.3 | 23.33 |
| 6 | 2 | 22 | 23.3 | 22.79 |
| 6 | 3 | 22 | 23.3 | 22.35 |
| 6 | 4 | 22 | 23.3 | 21.72 |
| 6 | 5 | 22 | 23.3 | 20.71 |
| 6 | 6 | 22 | 23.3 | 20.14 |
| 6 | 7 | 22 | 23.3 | 19.35 |
| 6 | 8 | 22 | 23.3 | 18.83 |
| 6 | 9 | 22 | 23.3 | 18.19 |
| 6 | 10 | 22 | 23.3 | 17.38 |
| 6 | 11 | 22 | 23.3 | 16 |
| 6 | 12 | 22 | 23.3 | 15.39 |
| 6 | 13 | 22 | 23.3 | 14.52 |
| 6 | 14 | 22 | 23.3 | 13.56 |
| 6 | 15 | 22 | 23.3 | 12.57 |
| 6 | 16 | 22 | 23.3 | 11.32 |
| 6 | 17 | 22 | 23.3 | 10.49 |
| 6 | 18 | 22 | 23.3 | 8.59 |
| 6 | 19 | 22 | 23.3 | 6.81 |
| 6 | 20 | 22 | 23.3 | 5.16 |
| 6 | 21 | 22 | 23.3 | 2.9 |
| 7 | 0 | 19.9 | 19.4 | 23.82 |
| 7 | 1 | 19.9 | 19.4 | 19.68 |
| 7 | 1.3 | 19.9 | 19.4 | 19.44 |
| 7 | 2 | 19.9 | 19.4 | 19.2 |
| 7 | 3 | 19.9 | 19.4 | 18.69 |
| 7 | 4 | 19.9 | 19.4 | 18.08 |
| 7 | 5 | 19.9 | 19.4 | 17.48 |
| 7 | 6 | 19.9 | 19.4 | 16.66 |
| 7 | 7 | 19.9 | 19.4 | 15.54 |
| 7 | 8 | 19.9 | 19.4 | 13.72 |
| 7 | 9 | 19.9 | 19.4 | 13.64 |
| 7 | 10 | 19.9 | 19.4 | 13.22 |
| 7 | 11 | 19.9 | 19.4 | 12.26 |
| 7 | 12 | 19.9 | 19.4 | 11.33 |
| 7 | 13 | 19.9 | 19.4 | 10.23 |
| 7 | 14 | 19.9 | 19.4 | 9.28 |
| 7 | 15 | 19.9 | 19.4 | 8.44 |
| 7 | 16 | 19.9 | 19.4 | 7.28 |
| 7 | 17 | 19.9 | 19.4 | 6.07 |
| 7 | 18 | 19.9 | 19.4 | 4.6 |
| 7 | 19 | 19.9 | 19.4 | 2.82 |
| 8 | 0 | 13.6 | 12.4 | 15.4 |
| 8 | 1 | 13.6 | 12.4 | 12.79 |
| 8 | 1.3 | 13.6 | 12.4 | 12.4 |
| 8 | 2 | 13.6 | 12.4 | 12.06 |
| 8 | 3 | 13.6 | 12.4 | 11.36 |
| 8 | 4 | 13.6 | 12.4 | 10.6 |
| 8 | 5 | 13.6 | 12.4 | 9.8 |
| 8 | 6 | 13.6 | 12.4 | 9.08 |
| 8 | 7 | 13.6 | 12.4 | 8.38 |
| 8 | 8 | 13.6 | 12.4 | 7.38 |
| 8 | 9 | 13.6 | 12.4 | 6.25 |
| 8 | 10 | 13.6 | 12.4 | 5.4 |
| 8 | 11 | 13.6 | 12.4 | 4.58 |
| 8 | 12 | 13.6 | 12.4 | 2.9 |
| 9 | 0 | 11.5 | 13.7 | 14.92 |
| 9 | 1 | 11.5 | 13.7 | 13.75 |
| 9 | 1.3 | 11.5 | 13.7 | 13.69 |
| 9 | 2 | 11.5 | 13.7 | 12.99 |
| 9 | 3 | 11.5 | 13.7 | 11.94 |
| 9 | 4 | 11.5 | 13.7 | 11.26 |
| 9 | 5 | 11.5 | 13.7 | 10.08 |
| 9 | 6 | 11.5 | 13.7 | 9.5 |
| 9 | 7 | 11.5 | 13.7 | 7.78 |
| 9 | 8 | 11.5 | 13.7 | 6.69 |
| 9 | 9 | 11.5 | 13.7 | 5.31 |
| 9 | 10 | 11.5 | 13.7 | 3.34 |
| 9 | 11 | 11.5 | 13.7 | 1.1 |
| 10 | 0 | 15 | 14.7 | 17.12 |
| 10 | 1 | 15 | 14.7 | 14.37 |
| 10 | 1.3 | 15 | 14.7 | 14.69 |
| 10 | 2 | 15 | 14.7 | 14.02 |
| 10 | 3 | 15 | 14.7 | 13.24 |
| 10 | 4 | 15 | 14.7 | 12.31 |
| 10 | 5 | 15 | 14.7 | 11.82 |
| 10 | 6 | 15 | 14.7 | 11.35 |
| 10 | 7 | 15 | 14.7 | 10.52 |
| 10 | 8 | 15 | 14.7 | 9.94 |
| 10 | 9 | 15 | 14.7 | 9.21 |
| 10 | 10 | 15 | 14.7 | 8.59 |
| 10 | 11 | 15 | 14.7 | 7.31 |
| 10 | 12 | 15 | 14.7 | 6.08 |
| 10 | 13 | 15 | 14.7 | 4.2 |
| 10 | 14 | 15 | 14.7 | 2.35 |
| 11 | 0 | 16.6 | 15.1 | 19.19 |
| 11 | 1 | 16.6 | 15.1 | 14.96 |
| 11 | 1.3 | 16.6 | 15.1 | 15.11 |
| 11 | 2 | 16.6 | 15.1 | 14.59 |
| 11 | 3 | 16.6 | 15.1 | 14.03 |
| 11 | 4 | 16.6 | 15.1 | 13.52 |
| 11 | 5 | 16.6 | 15.1 | 12.54 |
| 11 | 6 | 16.6 | 15.1 | 11.87 |
| 11 | 7 | 16.6 | 15.1 | 11.56 |
| 11 | 8 | 16.6 | 15.1 | 10.79 |
| 11 | 9 | 16.6 | 15.1 | 10.11 |
| 11 | 10 | 16.6 | 15.1 | 9.21 |
| 11 | 11 | 16.6 | 15.1 | 8.77 |
| 11 | 12 | 16.6 | 15.1 | 7.73 |
| 11 | 13 | 16.6 | 15.1 | 6.35 |
| 11 | 14 | 16.6 | 15.1 | 4.75 |
| 11 | 15 | 16.6 | 15.1 | 3.59 |
| 12 | 0 | 20.7 | 18.1 | 20.9 |
| 12 | 1 | 20.7 | 18.1 | 18.1 |
| 12 | 1.3 | 20.7 | 18.1 | 18.08 |
| 12 | 2 | 20.7 | 18.1 | 17.74 |
| 12 | 3 | 20.7 | 18.1 | 17.04 |
| 12 | 4 | 20.7 | 18.1 | 16.14 |
| 12 | 5 | 20.7 | 18.1 | 16.06 |
| 12 | 6 | 20.7 | 18.1 | 14.57 |
| 12 | 7 | 20.7 | 18.1 | 14.15 |
| 12 | 8 | 20.7 | 18.1 | 12.63 |
| 12 | 9 | 20.7 | 18.1 | 12.6 |
| 12 | 10 | 20.7 | 18.1 | 11.91 |
| 12 | 11 | 20.7 | 18.1 | 11.5 |
| 12 | 12 | 20.7 | 18.1 | 10.47 |
| 12 | 13 | 20.7 | 18.1 | 9.81 |
| 12 | 14 | 20.7 | 18.1 | 9.01 |
| 12 | 15 | 20.7 | 18.1 | 8.49 |
| 12 | 16 | 20.7 | 18.1 | 6.9 |
| 12 | 17 | 20.7 | 18.1 | 5.95 |
| 12 | 18 | 20.7 | 18.1 | 3.56 |
| 12 | 19 | 20.7 | 18.1 | 2.8 |
| 13 | 0 | 25 | 21.7 | 24.72 |
| 13 | 1 | 25 | 21.7 | 21.85 |
| 13 | 1.3 | 25 | 21.7 | 21.71 |
| 13 | 2 | 25 | 21.7 | 21.15 |
| 13 | 3 | 25 | 21.7 | 20.59 |
| 13 | 4 | 25 | 21.7 | 19.98 |
| 13 | 5 | 25 | 21.7 | 19.7 |
| 13 | 6 | 25 | 21.7 | 19.06 |
| 13 | 7 | 25 | 21.7 | 18.45 |
| 13 | 8 | 25 | 21.7 | 17.98 |
| 13 | 9 | 25 | 21.7 | 17.13 |
| 13 | 10 | 25 | 21.7 | 16.84 |
| 13 | 11 | 25 | 21.7 | 15.79 |
| 13 | 12 | 25 | 21.7 | 15.33 |
| 13 | 13 | 25 | 21.7 | 14.33 |
| 13 | 14 | 25 | 21.7 | 14.27 |
| 13 | 15 | 25 | 21.7 | 13.81 |
| 13 | 16 | 25 | 21.7 | 12.57 |
| 13 | 17 | 25 | 21.7 | 11.46 |
| 13 | 18 | 25 | 21.7 | 10.31 |
| 13 | 19 | 25 | 21.7 | 9.46 |
| 13 | 20 | 25 | 21.7 | 8.46 |
| 13 | 21 | 25 | 21.7 | 7.64 |
| 13 | 22 | 25 | 21.7 | 5.9 |
| 13 | 23 | 25 | 21.7 | 4.73 |
| 14 | 0 | 22.3 | 26.5 | 29 |
| 14 | 1 | 22.3 | 26.5 | 26.56 |
| 14 | 1.3 | 22.3 | 26.5 | 26.45 |
| 14 | 2 | 22.3 | 26.5 | 25.74 |
| 14 | 3 | 22.3 | 26.5 | 24.65 |
| 14 | 4 | 22.3 | 26.5 | 23.83 |
| 14 | 5 | 22.3 | 26.5 | 22.53 |
| 14 | 6 | 22.3 | 26.5 | 21.71 |
| 14 | 7 | 22.3 | 26.5 | 20.68 |
| 14 | 8 | 22.3 | 26.5 | 19.79 |
| 14 | 9 | 22.3 | 26.5 | 18.83 |
| 14 | 10 | 22.3 | 26.5 | 17.72 |
| 14 | 11 | 22.3 | 26.5 | 17.34 |
| 14 | 12 | 22.3 | 26.5 | 16.25 |
| 14 | 13 | 22.3 | 26.5 | 14.24 |
| 14 | 14 | 22.3 | 26.5 | 13.45 |
| 14 | 15 | 22.3 | 26.5 | 11.08 |
| 14 | 16 | 22.3 | 26.5 | 10.95 |
| 14 | 17 | 22.3 | 26.5 | 9.68 |
| 14 | 18 | 22.3 | 26.5 | 9.71 |
| 14 | 19 | 22.3 | 26.5 | 7.72 |
| 14 | 20 | 22.3 | 26.5 | 4.96 |
| 14 | 21 | 22.3 | 26.5 | 3.05 |
| 14 | 22 | 22.3 | 26.5 | 1.46 |
| 15 | 0 | 19.7 | 17.2 | 19.71 |
| 15 | 1 | 19.7 | 17.2 | 17.72 |
| 15 | 1.3 | 19.7 | 17.2 | 17.21 |
| 15 | 2 | 19.7 | 17.2 | 16.61 |
| 15 | 3 | 19.7 | 17.2 | 15.72 |
| 15 | 4 | 19.7 | 17.2 | 15.13 |
| 15 | 5 | 19.7 | 17.2 | 14.47 |
| 15 | 6 | 19.7 | 17.2 | 14.02 |
| 15 | 7 | 19.7 | 17.2 | 13.12 |
| 15 | 8 | 19.7 | 17.2 | 11.54 |
| 15 | 9 | 19.7 | 17.2 | 10.93 |
| 15 | 10 | 19.7 | 17.2 | 9.88 |
| 15 | 11 | 19.7 | 17.2 | 9.25 |
| 15 | 12 | 19.7 | 17.2 | 8.09 |
| 15 | 13 | 19.7 | 17.2 | 7.18 |
| 15 | 14 | 19.7 | 17.2 | 6.22 |
| 15 | 15 | 19.7 | 17.2 | 5.91 |
| 15 | 16 | 19.7 | 17.2 | 4.82 |
| 15 | 17 | 19.7 | 17.2 | 3.38 |
| 16 | 0 | 20.4 | 21.1 | 25.41 |
| 16 | 1 | 20.4 | 21.1 | 21.49 |
| 16 | 1.3 | 20.4 | 21.1 | 21.07 |
| 16 | 2 | 20.4 | 21.1 | 20.78 |
| 16 | 3 | 20.4 | 21.1 | 20.47 |
| 16 | 4 | 20.4 | 21.1 | 19.71 |
| 16 | 5 | 20.4 | 21.1 | 18.73 |
| 16 | 6 | 20.4 | 21.1 | 18.23 |
| 16 | 7 | 20.4 | 21.1 | 17.27 |
| 16 | 8 | 20.4 | 21.1 | 16.92 |
| 16 | 9 | 20.4 | 21.1 | 16.54 |
| 16 | 10 | 20.4 | 21.1 | 15.74 |
| 16 | 11 | 20.4 | 21.1 | 15.13 |
| 16 | 12 | 20.4 | 21.1 | 13.91 |
| 16 | 13 | 20.4 | 21.1 | 13.36 |
| 16 | 14 | 20.4 | 21.1 | 11.81 |
| 16 | 15 | 20.4 | 21.1 | 11.18 |
| 16 | 16 | 20.4 | 21.1 | 10 |
| 16 | 17 | 20.4 | 21.1 | 7.51 |
| 16 | 18 | 20.4 | 21.1 | 6.17 |
| 16 | 19 | 20.4 | 21.1 | 3.59 |
| 16 | 20 | 20.4 | 21.1 | 1.49 |
| 17 | 0 | 19.4 | 17.1 | 19.58 |
| 17 | 1 | 19.4 | 17.1 | 17.37 |
| 17 | 1.3 | 19.4 | 17.1 | 17.12 |
| 17 | 2 | 19.4 | 17.1 | 16.2 |
| 17 | 3 | 19.4 | 17.1 | 15.53 |
| 17 | 4 | 19.4 | 17.1 | 14.85 |
| 17 | 5 | 19.4 | 17.1 | 13.98 |
| 17 | 6 | 19.4 | 17.1 | 13.41 |
| 17 | 7 | 19.4 | 17.1 | 12.7 |
| 17 | 8 | 19.4 | 17.1 | 12.13 |
| 17 | 9 | 19.4 | 17.1 | 11.37 |
| 17 | 10 | 19.4 | 17.1 | 10.52 |
| 17 | 11 | 19.4 | 17.1 | 9.71 |
| 17 | 12 | 19.4 | 17.1 | 9.12 |
| 17 | 13 | 19.4 | 17.1 | 8.13 |
| 17 | 14 | 19.4 | 17.1 | 7.29 |
| 17 | 15 | 19.4 | 17.1 | 6.23 |
| 17 | 16 | 19.4 | 17.1 | 5.22 |
| 17 | 17 | 19.4 | 17.1 | 4.17 |
| 17 | 18 | 19.4 | 17.1 | 2.75 |
| 18 | 0 | 4.6 | 5.7 | 8.8 |
| 18 | 1 | 4.6 | 5.7 | 6.12 |
| 18 | 1.3 | 4.6 | 5.7 | 5.71 |
| 18 | 2 | 4.6 | 5.7 | 4.34 |
| 18 | 3 | 4.6 | 5.7 | 3.19 |
| 18 | 4 | 4.6 | 5.7 | 1.7 |
| 19 | 0 | 5.8 | 6.3 | 9.2 |
| 19 | 1 | 5.8 | 6.3 | 6.56 |
| 19 | 1.3 | 5.8 | 6.3 | 6.34 |
| 19 | 2 | 5.8 | 6.3 | 5.5 |
| 19 | 3 | 5.8 | 6.3 | 4.38 |
| 19 | 4 | 5.8 | 6.3 | 3.13 |
| 19 | 5 | 5.8 | 6.3 | 1.51 |
| 19 | 1.5 | 5.8 | 6.3 | 6.2 |
| 19 | 2.9 | 5.8 | 6.3 | 4.8 |
| 19 | 4.4 | 5.8 | 6.3 | 3 |
| 20 | 0 | 4.1 | 4.9 | 6.48 |
| 20 | 1 | 4.1 | 4.9 | 5.17 |
| 20 | 1.3 | 4.1 | 4.9 | 4.9 |
| 20 | 2 | 4.1 | 4.9 | 3.71 |
| 20 | 3 | 4.1 | 4.9 | 2.42 |
| 20 | 4 | 4.1 | 4.9 | 0.65 |
| 22 | 22.1 | 22.8 | 26.5 | 1.95 |
| 22 | 17 | 22.8 | 26.5 | 9.9 |
| 22 | 1.3 | 22.8 | 26.5 | 26.5 |
| 22 | 11.2 | 22.8 | 26.5 | 14.85 |
| 23 | 5 | 20 | 21.2 | 19.1 |
| 23 | 10 | 20 | 21.2 | 16.3 |
| 23 | 15 | 20 | 21.2 | 11.7 |
| 23 | 1.3 | 20 | 21.2 | 21.2 |
| 24 | 0 | 12.7 | 13.2 | 17.98 |
| 24 | 1 | 12.7 | 13.2 | 13.7 |
| 24 | 1.3 | 12.7 | 13.2 | 13.24 |
| 24 | 2 | 12.7 | 13.2 | 12.82 |
| 24 | 3 | 12.7 | 13.2 | 12.12 |
| 24 | 4 | 12.7 | 13.2 | 11.68 |
| 24 | 5 | 12.7 | 13.2 | 11.28 |
| 24 | 6 | 12.7 | 13.2 | 10.19 |
| 24 | 7 | 12.7 | 13.2 | 9.26 |
| 24 | 8 | 12.7 | 13.2 | 8.1 |
| 24 | 9 | 12.7 | 13.2 | 7.51 |
| 24 | 10 | 12.7 | 13.2 | 5.91 |
| 24 | 11 | 12.7 | 13.2 | 4.53 |
| 24 | 12 | 12.7 | 13.2 | 3.47 |
| 25 | 0 | 15.9 | 15.6 | 18.51 |
| 25 | 1 | 15.9 | 15.6 | 15.78 |
| 25 | 1.3 | 15.9 | 15.6 | 15.64 |
| 25 | 2 | 15.9 | 15.6 | 15.53 |
| 25 | 3.6 | 15.9 | 15.6 | 14.71 |
| 25 | 5.6 | 15.9 | 15.6 | 13.35 |
| 25 | 9.6 | 15.9 | 15.6 | 9.45 |
| 25 | 11.6 | 15.9 | 15.6 | 6.65 |
| 25 | 13.6 | 15.9 | 15.6 | 3.31 |
| 25 | 15 | 15.9 | 15.6 | 0.97 |
| 26 | 1.3 | 7 | 9.5 | 9.5 |
| 26 | 1.8 | 7 | 9.5 | 9.1 |
| 26 | 3.5 | 7 | 9.5 | 6.8 |
| 26 | 5.3 | 7 | 9.5 | 4 |
| 27 | 1.3 | 13.8 | 14.7 | 14.7 |
| 27 | 3.5 | 13.8 | 14.7 | 13.1 |
| 27 | 6.9 | 13.8 | 14.7 | 10.9 |
| 27 | 10.4 | 13.8 | 14.7 | 7.3 |
| 28 | 1.3 | 15 | 12.3 | 12.3 |
| 28 | 3.7 | 15 | 12.3 | 11.2 |
| 28 | 7.5 | 15 | 12.3 | 9 |
| 28 | 11.2 | 15 | 12.3 | 5.9 |
| 29 | 1.3 | 11.2 | 11.4 | 11.4 |
| 29 | 2.8 | 11.2 | 11.4 | 10.1 |
| 29 | 5.6 | 11.2 | 11.4 | 8.1 |
| 29 | 8.4 | 11.2 | 11.4 | 4.9 |
| 30 | 0 | 9.6 | 10.9 | 12.85 |
| 30 | 1 | 9.6 | 10.9 | 10.97 |
| 30 | 1.3 | 9.6 | 10.9 | 10.86 |
| 30 | 2 | 9.6 | 10.9 | 10.29 |
| 30 | 3 | 9.6 | 10.9 | 9.43 |
| 30 | 4 | 9.6 | 10.9 | 8.81 |
| 30 | 5 | 9.6 | 10.9 | 7.27 |
| 30 | 6 | 9.6 | 10.9 | 6.26 |
| 30 | 7 | 9.6 | 10.9 | 4.92 |
| 30 | 8 | 9.6 | 10.9 | 3.18 |
| 30 | 9 | 9.6 | 10.9 | 0.96 |
| 31 | 0 | 10.5 | 11 | 13.76 |
| 31 | 1 | 10.5 | 11 | 11.21 |
| 31 | 1.3 | 10.5 | 11 | 11.04 |
| 31 | 2 | 10.5 | 11 | 10.76 |
| 31 | 3 | 10.5 | 11 | 9.81 |
| 31 | 4 | 10.5 | 11 | 9.07 |
| 31 | 5 | 10.5 | 11 | 8.53 |
| 31 | 6 | 10.5 | 11 | 7.54 |
| 31 | 7 | 10.5 | 11 | 6.51 |
| 31 | 8 | 10.5 | 11 | 5.89 |
| 31 | 9 | 10.5 | 11 | 4.06 |
| 31 | 10 | 10.5 | 11 | 2.8 |
| 32 | 0 | 13.8 | 19.5 | 22.68 |
| 32 | 1 | 13.8 | 19.5 | 20.96 |
| 32 | 1.3 | 13.8 | 19.5 | 19.52 |
| 32 | 2 | 13.8 | 19.5 | 18.66 |
| 32 | 3 | 13.8 | 19.5 | 17.61 |
| 32 | 4 | 13.8 | 19.5 | 16.55 |
| 32 | 5 | 13.8 | 19.5 | 15.27 |
| 32 | 6 | 13.8 | 19.5 | 14 |
| 32 | 7 | 13.8 | 19.5 | 12.49 |
| 32 | 8 | 13.8 | 19.5 | 10.52 |
| 32 | 9 | 13.8 | 19.5 | 8.62 |
| 32 | 10 | 13.8 | 19.5 | 7.17 |
| 32 | 11 | 13.8 | 19.5 | 5.86 |
| 32 | 12 | 13.8 | 19.5 | 3.45 |
| 32 | 13 | 13.8 | 19.5 | 2.38 |
| 33 | 0 | 15.5 | 10.4 | 15.69 |
| 33 | 1 | 15.5 | 10.4 | 10.85 |
| 33 | 1.3 | 15.5 | 10.4 | 10.4 |
| 33 | 2 | 15.5 | 10.4 | 9.21 |
| 33 | 5.6 | 15.5 | 10.4 | 7.7 |
| 33 | 8.6 | 15.5 | 10.4 | 6.45 |
| 33 | 11.6 | 15.5 | 10.4 | 5 |
| 33 | 14.6 | 15.5 | 10.4 | 3.73 |
| 34 | 5 | 24.5 | 28.4 | 25.33 |
| 34 | 6 | 24.5 | 28.4 | 24.34 |
| 34 | 8 | 24.5 | 28.4 | 23.02 |
| 34 | 10 | 24.5 | 28.4 | 22.07 |
| 34 | 11 | 24.5 | 28.4 | 21.69 |
| 34 | 12 | 24.5 | 28.4 | 21.24 |
| 34 | 13 | 24.5 | 28.4 | 20.38 |
| 34 | 14 | 24.5 | 28.4 | 19.11 |
| 34 | 16 | 24.5 | 28.4 | 17.58 |
| 34 | 18 | 24.5 | 28.4 | 16.47 |
| 34 | 19 | 24.5 | 28.4 | 15.54 |
| 34 | 20 | 24.5 | 28.4 | 13.82 |
| 34 | 21 | 24.5 | 28.4 | 11.95 |
| 34 | 22 | 24.5 | 28.4 | 10.66 |
| 34 | 23 | 24.5 | 28.4 | 7.66 |
| 34 | 24 | 24.5 | 28.4 | 3.82 |
| 35 | 1 | 25.5 | 15.8 | 15.99 |
| 35 | 1.3 | 25.5 | 15.8 | 15.78 |
| 35 | 2 | 25.5 | 15.8 | 15.64 |
| 35 | 4 | 25.5 | 15.8 | 15.53 |
| 35 | 7 | 25.5 | 15.8 | 14.71 |
| 35 | 10 | 25.5 | 15.8 | 13.35 |
| 35 | 13 | 25.5 | 15.8 | 11.43 |
| 35 | 16 | 25.5 | 15.8 | 9.45 |
| 35 | 19 | 25.5 | 15.8 | 6.65 |
| 35 | 22 | 25.5 | 15.8 | 3.31 |
| 35 | 25 | 25.5 | 15.8 | 0.97 |
