# Supplementary material for: A Mixed-Effects Model with Different Strategies for Modeling Volume in Cunninghamia lanceolata Plantations
Source: PLoS One. 2015 Oct 7;10(10):e0140095. doi: 10.1371/journal.pone.0140095 (PMC4596836; doi:10.1371/journal.pone.0140095)
Supplement: S2 Text — (DOCX) [file pone.0140095.s002.docx]

x=read.table("DATA.txt",header=T);attach(x);library(nlme)

f.nls=nls(d~2*((b0*D)/(1-exp(b2*(1.3-H)))+(D/2-b0*D)*(1-(1/(1-exp(b1*(1.3-H)))))+(exp(-b1*h))*(((D/2-b0*D)*exp(1.3*b1))/(1-exp(b1*(1.3-H))))-exp(b2*h)*((b0*D*exp(-b2*H))/(1-exp(b2*(1.3-H))))),data=x,start=list(b0=0.3435,b1=0.65,b2=0.09))

summary(f.nls)

n=463

m=2

p=predict(f.nls)

write.table((p),file="MGY.txt")

U=p

cc=U-d

jdcc=abs(cc)

E=sum(jdcc)/n

E

RMSE=sqrt(sum((cc)^2)/(n-1))

RMSE

d.mean=mean(d)

R2=1-((sum((cc)^2))/(sum((d-d.mean)^2)))

R2

AIC(f.nls)

BIC(f.nls)

logLik(f.nls)
